# Validated Computational Model to Compute Re-apposition Pressures for Treating Type-B Aortic Dissections

**DOI:** 10.3389/fphys.2018.00513

**Published:** 2018-05-09

**Authors:** Aashish Ahuja, Xiaomei Guo, Jillian N. Noblet, Joshua F. Krieger, Blayne Roeder, Stephan Haulon, Sean Chambers, Ghassan S. Kassab

**Affiliations:** ^1^California Medical Innovations Institute, San Diego, CA, United States; ^2^Cook Medical, Bloomington, IN, United States; ^3^Aortic Center, Hôpital Marie Lannelongue, Université Paris Sud, Paris, France

**Keywords:** aortic dissection, bench tests, porcine aorta, finite element analysis, re-apposition pressure, simulation models

## Abstract

The use of endovascular treatment in the thoracic aorta has revolutionized the clinical approach for treating Stanford type B aortic dissection. The endograft procedure is a minimally invasive alternative to traditional surgery for the management of complicated type-B patients. The endograft is first deployed to exclude the proximal entry tear to redirect blood flow toward the true lumen and then a stent graft is used to push the intimal flap against the false lumen (FL) wall such that the aorta is reconstituted by sealing the FL. Although endovascular treatment has reduced the mortality rate in patients compared to those undergoing surgical repair, more than 30% of patients who were initially successfully treated require a new endovascular or surgical intervention in the aortic segments distal to the endograft. One reason for failure of the repair is persistent FL perfusion from distal entry tears. This creates a patent FL channel which can be associated with FL growth. Thus, it is necessary to develop stents that can promote full re-apposition of the flap leading to complete closure of the FL. In the current study, we determine the radial pressures required to re-appose the mid and distal ends of a dissected porcine thoracic aorta using a balloon catheter under static inflation pressure. The same analysis is simulated using finite element analysis (FEA) models by incorporating the hyperelastic properties of porcine aortic tissues. It is shown that the FEA models capture the change in the radial pressures required to re-appose the intimal flap as a function of pressure. The predictions from the simulation models match closely the results from the bench experiments. The use of validated computational models can support development of better stents by calculating the proper radial pressures required for complete re-apposition of the intimal flap.

## Introduction

The incidence of aortic dissection (AD) in the United States is approximately 2,000 cases per year and early mortality is as high as 1% per hour if untreated (Vecht et al., 1980; Roberts, 1981). AD occurs when there is a tear in the intimal lining of the aorta, allowing blood to flow between the intimal and medial layers of the aorta. Complicated type-B acute AD is specifically defined as dissection associated with rupture, malperfusion syndromes, refractory pain, or rapid aortic expansion at onset or during hospital stay (Fattori et al., 2008). Currently, there are three modes of treating patients suffering from type-B acute AD: medical management, open surgery, or endovascular treatment. While medical management is suggested for patients who have uncomplicated dissections, open surgery or endovascular treatment is recommended for complicated dissections.

Recently, clinicians recommend the minimally invasive endovascular grafting procedure over open surgery for complicated type-B patients suffering from impending or actual complicated dissections because surgical methods have approximately a 13% higher mortality rate at 5-year follow-up as compared to endovascular grafting (Moulakakis et al., 2014). The endograft is first deployed to exclude the proximal entry tear to redirect blood flow toward the true lumen (TL) and then a stent or graft is used to push the intimal flap against the false lumen (FL) wall such that the aorta is reconstituted by sealing the FL. Although stent grafting has been largely successful in closing the initial entry tear (Kato et al., 2002; Song et al., 2006; Marcheix et al., 2008; Sayer et al., 2008; Alves et al., 2009; Manning et al., 2009; Czerny et al., 2010), patients may still undergo re-interventions in the form of multiple stent-grafts or open surgery due to ongoing complications (Thrumurthy et al., 2011; Fattori et al., 2013; Andersen et al., 2014; Faure et al., 2014). Aortic aneurysm is one of the complications that develops in the FL wall after successful endovascular treatments. The occurrence of aneurysm in patients with successful treatments is reported to be about 7% (Won et al., 2006)–20% (Lopera et al., 2003), which is likely attributed to perturbation of significant mechanical forces on the aorta (Kassab, 2006; Fortier et al., 2014). Moreover, a significant enlargement of the aortic diameter above the stent graft has been observed during deployment, which is important because this may reflect increased strain and stress on the aorta in the segments adjacent to the stent graft (Trimarchi and Eagle, 2016).

The observation of aortic aneurysm in the FL after the stent graft procedure illustrates the necessity of fine-tuning the procedure to minimize the risk for the patient of reintervention after the initial endovascular graft. Using a detailed and systematic biomechanical analysis of blood pressure in both the mid and distal regions of the TL, may lead to the design and use of proper endovascular grafts or bare metal stents which provides sufficient radial forces on the intimal flap to re-appose it against the FL wall. This may allow reconstitution of the aortic wall without imposing high mechanical stresses on the FL wall, leading to a decrease in secondary complications such as aortic aneurysm. The development of effective mechanical devices for endovascular grafting requires the use of computational techniques such as Finite Element Analysis (FEA) to analyze the structural interaction between the rigid stents (usually composed of Stainless steel or Nitinol alloy) and different tissue segments of the dissected aorta (i.e., intimal flap, FL wall, and true lumen [TL] wall). FEA has been used in the past to compute radial forces and improve stent designs being deployed for treatment of stenosed valves (Kumar and Mathew, 2010), atherosclerotic coronary arteries (Eshghi et al., 2011) and aortic aneurysms (Arokiaraj et al., 2014). The present study utilized FEA utilized to develop a bench-validated computational model based on contact mechanics to quantify the radial pressures required to reconstitute the aorta from dissection and promote remodeling, without exerting undue strains on the vessel wall.

The purpose of this study is to provide a validated computational model of flap re-apposition which can be used as a benchmark for testing different therapies in a virtual setting. The proposed model aims to reduce the overall time and cost required to perform pre-clinical *in vivo* studies in porcine and expedite the development of better endovascular therapies for treating AD in humans. From the bench tests and computational simulations presented, it is observed that the radial pressure for re-apposition of intimal flap increases monotonically with increase in aortic pressure.

## Materials and Methods

### Bench Experiments

#### Aorta Preparation and Dissection

**FIGURE 1:**
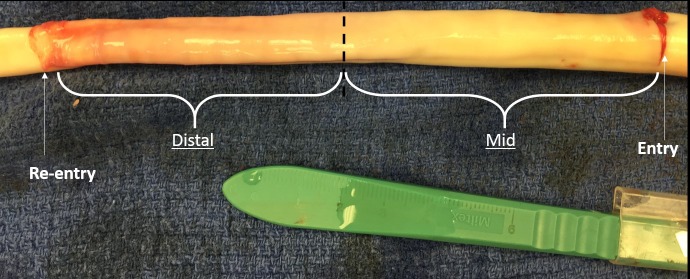
An inverted aorta with an imposed dissection. An entry was initially created in the descending thoracic aorta and propagated using forceps to the distal region of the aorta where a pocket of re-entry was created. Tissue specimens from two regions (mid and distal) were extracted and tested on planar biaxial testing machine for material characterization.

Five fresh thoracic aorta samples harvested from healthy porcine were prepared for bench tests by removing all connective tissue and ligating all branches using 2-0 sutures. Samples were stored at ∼2°C until tested. Samples were not used for any testing prior to this research. Next, the porcine aorta was inverted exposing the intima and dissections were created ∼6–8 cm from the left subclavian artery (LSA). The percent circumferential length of the entry tear, calculated as 100 × (circumference of the flap/circumference of the aorta), was approximately 50–60% of aorta circumference (Canchi et al., 2017). Dissections in healthy descending thoracic aorta represent the case of acute Type-B AD. Using a surgical blade, a cut was made in the inner lining of the aortic wall. The layers were separated using a surgical blade and advanced using fine-tip forceps to the desired axial length. The depth of incision for dissection was typically set between 30 and 50% of the wall thickness. A resulting intimal flap of about ∼10–13 cm in length was created due to surgical dissection as shown in **Figure 1**. A reentry was then created, and the flap separated the TL from the FL of the aorta.

#### Protocol for Bench Testing

**FIGURE 2:**
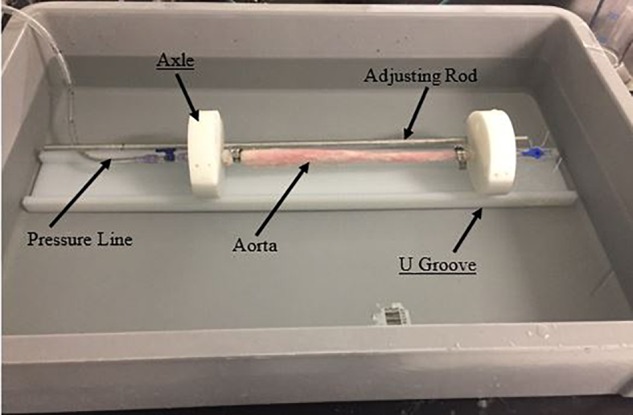
Fixture and setup for conducting bench tests on dissected aorta.

**FIGURE 3:**
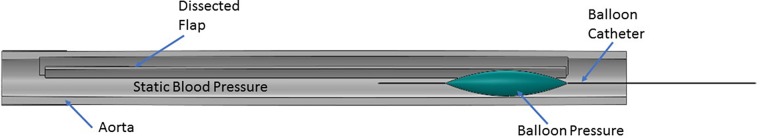
Representation of the protocol followed during the bench test. A balloon catheter was inserted into the true lumen (TL). The balloon was inflated with water which applied bending forces to either the mid or distal region of the flap in the presence of static aortic pressure. The balloon pressure on re-apposition of flap was recorded using a pressure transducer.

After the dissection was created in the aorta sample, it was mounted on a customized static pressure fixture developed for this study as shown in **Figure 2**. In the setup, the pressure line was connected to stopcock valve which was attached on the other end with an axle as shown in left side of image. The stopcock on right was always closed and to prevent outflow of saline. A saline water reservoir was fed with a compressed air line at a specific pressure and as a result, the pressure line was filled with saline water. The saline solution was transmitted to the suspended aorta which inflated under the applied pressure. Prior to performing the bench test, a Cook CODA balloon catheter (CODA-2-9.0-35-100-32) was advanced into, either the mid (∼6–8 cm from LSA) or distal (∼11–14 cm from LSA) region of the TL, such that the entire balloon was contained within the dissection. The aorta was then pressurized to static pressures between 70 and 140 mmHg in increments of 10 mmHg. Higher aortic pressures simulated patients with hypertension, which is considered one of the primary risk factors for AD. For each of the sample pressure values, the balloon was inflated by filling it with water until the intimal flap re-apposed against the outer wall. The reconstitution of the aortic wall was confirmed via ultrasound imaging (Philips iE33) and the corresponding pressure for balloon inflation was recorded using pressure transducer. The pressure for re-apposition was calculated as the difference between balloon and static aorta pressure; i.e., Δ (Balloon-Aortic pressure). **Figure 3** shows the schematic of bench test.

### Finite Element Modeling of Dissected Aorta

#### Tissue Materials

Aortic dissection splits a single aortic lumen into two lumens: FL and TL. As a result, material properties for the dissected layers (i.e., FL wall and intimal flap), in addition to the undissected TL wall, are required to develop fully informed FEA models. As demonstrated in Ahuja et al. (unpublished) the material characterization for the mid and distal regions of the dissected layers, FL wall, and intimal flap, were calculated first by, conducting planar biaxial testing on porcine tissue specimens and then, using a non-linear regression algorithm (Nelder-Mead optimization method; Nelder and Mead, 1965) to fit experimental data against a structural constitutive model (Gasser et al., 2006). Details of the material characterization of tissue samples including their measured experimental curves with model fitting are shown in Appendix 1.

The tissue components of the dissected aorta behave as a hyperelastic material. Since the intimal flap undergoes large bending stresses due to distension of the expansion member, different material properties are applied in the definition of both mid and distal flap segments. Using each of the porcine aortas, we created five sets of parameters for mid flap segments and four sets of parameters for distal flap segments. Thus, a total of nine different flap geometries were created using computer aided design (CAD) software. One set of material definitions for descending thoracic TL wall, mid FL wall and distal FL wall were used throughout the simulations. **Table 1** gives the parameter estimates for the mid and distal flaps and **Table 2** gives the parameter values for the TL wall, Mid FL wall, and Distal FL wall.

**Table 1:** Parameter estimation for mid and distal flap.

Pig #	Thickness (mm)	C10 (Pa)	k1 (Pa)	k2	α (degrees)	κ
**Parameter estimation for mid flap**		
1	0.58	92,963	230,290	13.9	87.1	0.33
2	0.59	73,144	235,075	7.86	68.7	0.3
3	0.54	64,042	212,120	4.99	23.5	0.32
4	0.70	52,072	125,430	5.87	53.9	0.26
5	0.47	45,588	149,880	1.42	55.6	0.21
**Parameter estimation for distal flap**		
1	0.4	103,140	61,969	4.1	62.4	0.1
2	0.34	171,740	661,830	8.05	86.5	0.3
3	0.43	78,686	239,090	3.18	89.9	0.3
4	0.29	63,554	77,013	4.76	83.4	0.11

**Table 2:** Parameter estimation for TL wall, mid FL wall, and distal FL wall.

Parameter estimation for mid flap						
Region	Thickness (mm)	C10 (Pa)	k1 (Pa)	k2	α (degrees)	κ
TL wall	1.76	78,219	201,440	1.52	87.1	0.2
Mid FL wall	1.3	53,456	952,380	4.94	7.5	0.3
Distal FL wall	1.24	72,996	20,894	9.01	66.5	0

#### Pre-processing

**FIGURE 4:**
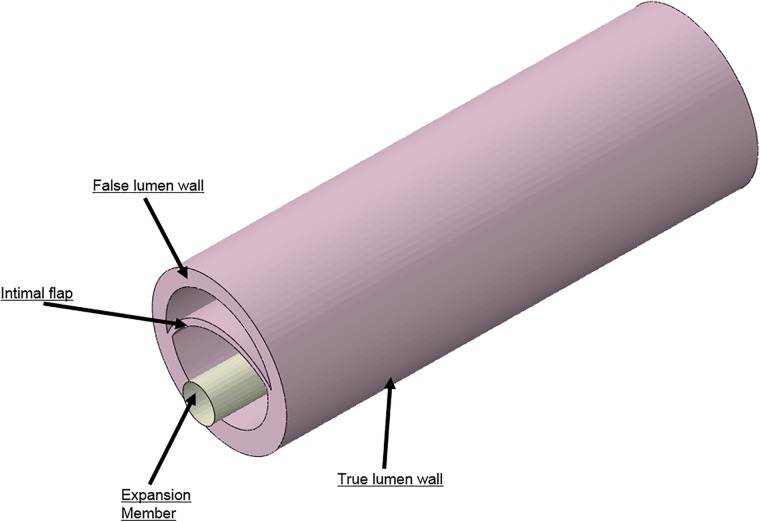
Different geometries required to simulate aortic dissection are developed as 3D computer aided design (CAD) models and imported into finite element analysis (FEA) software.

Idealized models of a dissected aorta were prepared using Solidworks (v2017, Dassault Systemes) and simulated in Abaqus (v6.13.5, Dassault Systemes) using the Abaqus CAE (Complete Abaqus Environment) standard module. The 3D CAD geometry for the porcine aorta is shown in **Figure 4** which consisted of three layers; i.e., TL wall, FL wall and intimal flap. The circumferential length of the intimal flap was adjusted to be within 50–60% of the aortic circumferential length. A rigid expansion member was modeled within the TL of the dissected aorta to re-appose the intimal flap by expansion, which is analogous to the inflation of a balloon.

**FIGURE 5:**
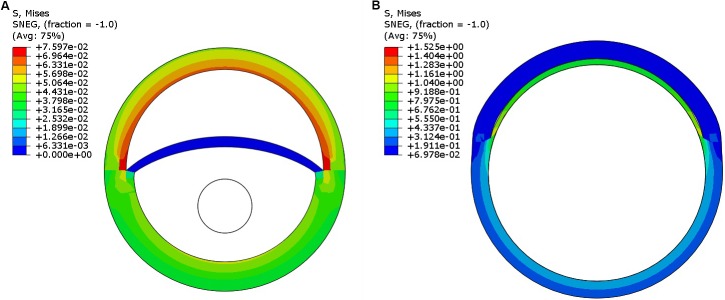
**(A)** Pressurization of the dissected aorta to an internal pressure of 100 *mmHg.* **(B)** Expansion member was distended to achieve full re-apposition of the intimal flap.

All the components of the model were meshed using hexahedral elements (C3D8RH), except the expansion member, which was meshed using linear shell elements (S4). The intimal flap underwent bending due to contact force being applied by the expansion member which was meshed with at least two rows of C3D8RH elements to avoid hourglass effects. Three contact interactions were established between the following: (1) the expansion member and intimal flap, (2) the expansion member and TL wall, and (3) the intimal flap and FL wall. The simulation for re-apposition of the intimal flap was conducted over two steps to match the protocol followed for bench tests:

Step 1: Pressurize the dissected aorta to the required internal pressure (70–140 mmHg) (**Figure 5A**)
Step 2: While maintaining the aorta in a pressurized state (from Step 1), distend the expansion member and achieve full re-apposition of the intimal flap (**Figure 5B**).

Four boundary conditions (BCs) and one loading condition (LC) were applied:

BC 1: Constrained the z-deformation of vessel end faces.
BC 2: Constrained the nodes lying on YZ-plane passing through center of dissected aorta (X-symmetry).
BC 3: Constrained the nodes on faces containing flap, FL wall, and TL wall to move along the y-direction.
BC 4: Dilated the expansion member using specified displacement in Step 2.
LC 1: Pressurized the interior of the aorta by applying pressure to selected areas in Step 1.

A sensitivity analysis on mesh was performed to show that the results converged to the same solution with a refined mesh as well. A global element size of 0.3 mm for both the dissected aorta and expansion member was selected for all the samples and cases considered. The results for radial pressures at 100 mmHg converged to the same solution within an error of 2.5% compared to a refined mesh. Since the thicknesses of flaps varied between computational models, mesh sensitivity results from Distal Sample #1 are presented here. The global element size of 0.3 mm resulted in 69,126 hexahedral elements for the dissected aorta model. The radial pressures from this model only decreased by 1.7% when compared to a model with a global element size of 0.2 mm (233,655 hexahedral elements). Also, choosing the latter finer mesh size increased computational time by an average of 200% (from 0.63 h to 1.9 h) when solved on *San Diego Supercomputer Center’s* (SDSC) workstation utilizing 24 cores (Towns et al., 2014).

#### Post Processing

The contact pressure applied to the expansion member due to interaction with the intimal flap and TL wall was computed for all aortic pressures between 70 and 140 mmHg. The average of the contact pressure over all elements of the expansion member returned the radial pressure, which was calculated using a customized Python script. The radial pressures computed for each static aorta pressure was compared with re-apposition pressure obtained from bench experiments.

## Results

### Re-apposition of Mid Flap Against the Mid FL Wall

**FIGURE 6:**
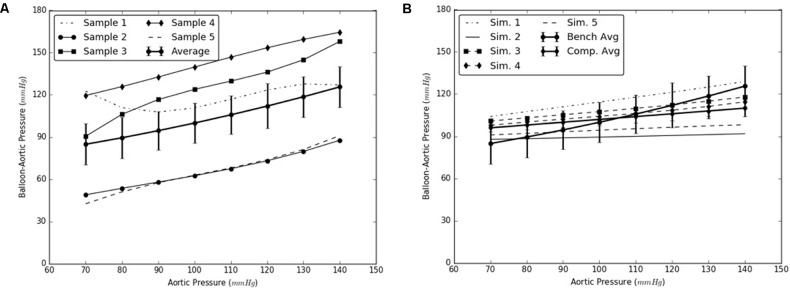
**(A)** Bench test measurements for re-apposition of mid flap against the mid false lumen (FL) wall. **(B)** Computational measurements for re-apposition of mid flap and its comparison with bench results.

Under this solution, the expansion member was distended to push the mid region of the intimal flap until it re-apposed completely against the mid FL wall. An increase in the diameter of the aorta was observed after re-apposition of intimal flap. During experiments, the static pressure was homogenously applied to the entire aorta vessel as well as to the expanding balloon. In the case of simulations, the aorta vessel was loaded with a pressure equal to that applied during bench testing, but the expansion member was unloaded. Hence, we define re-apposition pressure as P_re-app_ = P_balloon_ - P_aorta_ in the experimental results, while for simulations we obtain this value, P_re-app_ (simulation), directly from the pressure applied on the expansion member. The values of Δ (Balloon-Aortic pressure) with respect to static aortic pressures are shown in **Figures 6A,B**.

### Re-apposition of Distal Flap Against Distal FL Wall

**FIGURE 7:**
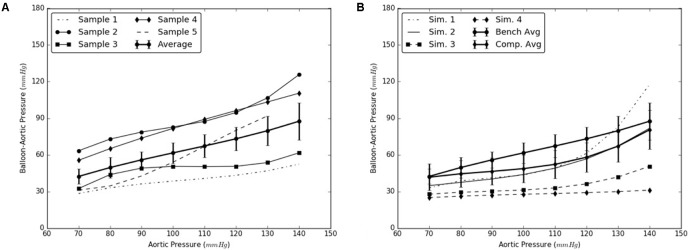
**(A)** Bench test measurements for re-apposition of distal flap against the distal false lumen (FL) wall. **(B)** Computational measurements for re-apposition of distal flap and its comparison with bench results.

Similarly, the expansion member was distended to push the distal region of the flap until it re-apposed completely against the distal FL wall. The radial pressures required for re-apposition were smaller than those required for re-apposition of mid flap. The results from bench tests and computational models are shown in **Figures 7A,B**, respectively.

### Change in Diameter of Aorta Due to Re-apposition

Results from the bench experiments showed an increase in the diameter of a dissected aorta with application of aortic pressure. Specifically, the diameter of the dissected aorta was affected by two parameters:

(1) An increase in pressure resulted in an overall increase in the diameter of the vessel
(2) An increase in diameter imposed additional tension on the flap which increased the net balloon pressure to re-appose the flap. This, in turn required a bigger balloon diameter to re-appose the flap completely against the wall.

**FIGURE 8:**
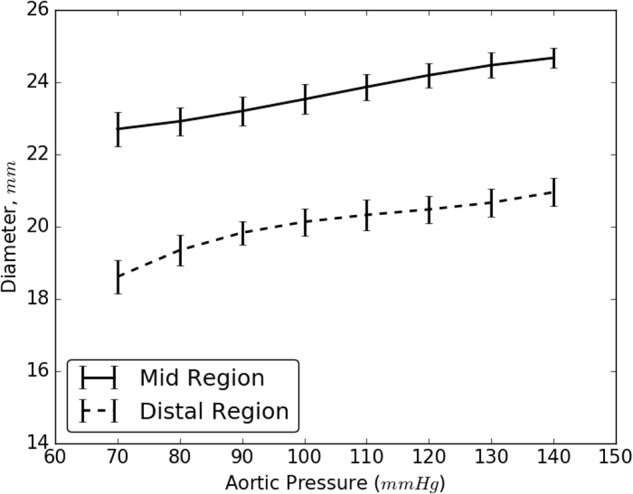
Resulting diameter of the mid and distal region of dissected aorta on complete re-apposition of intimal flap.

**FIGURE 9:**
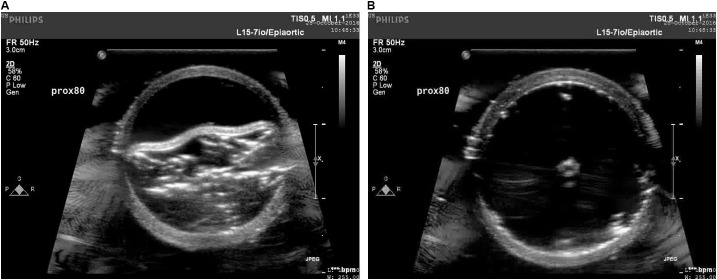
Ultrasound images capturing various stages: **(A)** crimped and **(B)** expanded, of a CODA balloon under aortic pressure of 80 mmHg.

The increase in the internal diameter of the aorta for all considered static pressures (i.e., 70–140 mmHg), for distal and mid region is shown in **Figure 8**. The values for the diameter of the vessel after re-apposition were measured from ultrasound images. The images were captured at the start of each bench experiment (when balloon was completely crimped) and at the end of expansion (when flap was in complete contact with false lumen wall). As an example, **Figure 9** shows the ultrasound images highlighting the expansion of CODA balloon under 80 mmHg aortic pressure. It should be noted that the diameter of the distal part of the aorta always had a smaller value than the mid region of the aorta, for all static pressures imposed during bench testing.

### Sensitivity Analysis

The flap, which is pre-stressed as part of the intact aorta, shortens after the dissection. It also undergoes large bending and stretching during the re-apposition process which also leads to further stiffening of the flap. Since the present study used material properties based on biaxial testing of different samples of intimal flap, sensitivity analyses were conducted on TL and FL walls for both mid and distal regions of the dissected aorta. It was observed that only the intimal flap largely influenced the radial pressures recorded during the re-apposition process. To conduct the sensitivity analysis, we varied the material properties of the distal FL wall, mid FL wall or TL(using Tables 2, 3, 5 from the Appendix). The material properties from Sample #1 were utilized for dissection components that are unchanged.

**FIGURE 10:**
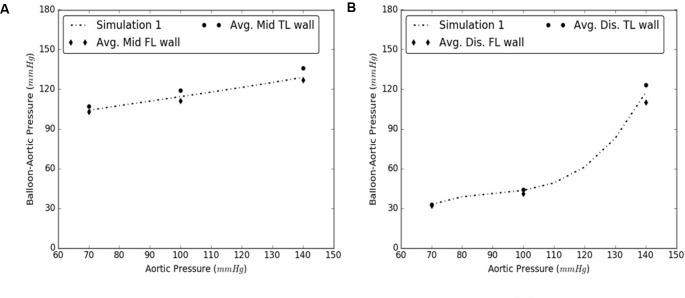
Comparison between results from Sample #1 and averages of samples #2-5 for **(A)** Mid, and **(B)** Distal dissections.

The simulations were executed for aortic pressures of 70, 100, and 140 mmHg. It was concluded that the radial pressures can be accurately predicted by varying material behavior of intimal flap while using only one set of TL and FL wall properties since the average results from Samples 2–5 are within 6 and 8% of the mid and distal computational results, respectively, observed for Sample #1. See **Figures 10A,B**.

## Discussion

Since the introduction of thoracic endovascular aortic repair (TEVAR) in Dake et al. (1999), Mitchell et al. (1999), TEVAR has gained popularity over surgical repair for treating complicated Stanford Type B aortic dissections. Many studies have shown favorable short- and mid-term outcomes for Type B dissections using TEVAR. In mid-term and long-term follow-ups, however, distal stent-related complications including distal re-entry, pseudoaneurysm formation, and aneurysm formation are observed (Neuhauser et al., 2008; Thrumurthy et al., 2011). Studies have reported that the incidence of new re-entries at the proximal and distal ends of the stent graft, combined are 3.4% (out of 651 patients) (Dong et al., 2010). In a sub-cohort of 23 patients with re-entries (3.4% of the 651 patients), 25% (4 of 16) were at the proximal end of the stent graft and 28.6% (2 of 7) were at the distal ends of the stent graft, leading to a total mortality rate of 23.1% of the sub-cohort. Most of the stent-related complications were caused by conventional stent grafts which likely imposed abnormally high stresses and strains on the aortic wall. The present study utilized FEA to develop a bench-validated computational model which quantified the exact radial pressures required to re-appose the flap after dissection. This will allow proper stents and grafts sizing, while avoiding oversizing to prevent undue strains and stresses on the aorta which can lead to retrograde dissection (Leshnower et al., 2013), in-folding, and graft occlusion (Kasirajan et al., 2012).

The use of this validated computational models can expedite the development of novel stents and therapies in treating aortic dissection. The model presented in this study uses a well-informed but idealized computer model of dissection in porcine, which captures the balloon pressures that are required to re-appose the intimal flap in reconstituting the aorta lumen. We developed two sets of models, one for mid dissections and one for and distal dissections, and presented results for radial pressures under static aortic pressures. From our plots in **Figure 6**, we observed that the computational models captured the bench results for all pressures, 70–140 mmHg. This included physiological blood pressures (mean pressure ∼90 mmHg) as well as high blood pressures. A small standard error (2.8 – 6.1 mmHg) between results from computational models and bench test measurements was observed and the slope for average curve was small. The radial pressures applied to stent grafts for re-apposition increased slowly in the case of mid region. Overall for distal region shown in **Figure 7**, the radial pressures required for re-apposition were lower than those observed in mid region, but also increased slowly. There were two reasons for lower radial pressure values: (1) The distal aorta had a smaller diameter as compared to mid aorta and thus the balloon or expansion member required less distension to re-appose the intimal flap, and (2) The distal flap underwent lower circumferential stretch as compared to mid flap and was hence, more compliant at those stretch values (Ahuja et al. unpublished). Finally, the average results from bench experiments were within the range for computational outputs for both mid and distal regions.

As the aortic pressure increased, we simultaneously saw an increase in the resultant diameter of the vessel following the re-apposition of intimal flap. The resulting diameters for both, mid and distal regions, increased monotonically and followed the same trend for all pressures, 70–140 mmHg. These curves, shown in **Figure 8**, had large slopes for pressures ≤110 mmHg, but became more horizontal at greater pressures. This behavior may be attributed to the presence of collagen fibers which became uncrimped and lead to stiffening of tissues at higher pressures. The fibers may have reduced the rate of dilation of the aorta and hence smaller changes were observed in the diameters at higher pressures.

### Limitations

The computational model for re-apposition considered an informed geometrically idealized model to validate the results from bench experiments. Further, we used different samples for bench and computational studies. A more accurate computational model based on one-to-one true geometry of the aorta would have generated results that correlated closer with those obtained from bench tests. An accurate model could be reconstructed from echocardiography by acquiring high resolution cross-sectional images for the entire aorta with a thickness of around 1 mm/slice (Ohbuchi and Fuchs, 1991). Despite the geometric idealization, our current model predictions were in very good agreement with the bench data.

Furthermore, current simulations considered balloon inflation through displacement boundary conditions. Realistic approaches for inflation of the balloon could be found in the literature (e.g., De Beule et al., 2008; Martin and Boyle, 2013; Schiavone and Zhao, 2015). Our current study relied on static aortic pressure to quantify the radial pressures required to re-appose the flap. In future models, we could correlate our computational results with *in vivo* studies using a better representation of folded balloon inflation, experiencing the dynamic conditions of aortic blood flow and pressure. In bench testing, we manually created dissections by first, initiating a tear and then using a surgical blade to advance the tear into a flap. This method leads to generation of a straight linear flap. *In vivo* dissections have shown the intimal flap to rotate inside the aorta as it progresses from the thoracic aorta to abdominal aorta (i.e., spiral dissections) (Armstrong et al., 1996). Also, the occurrence of dissection may lead to creation of multiple re-entries along the length of the intimal flap (Criado, 2011). These complicated dissections have been known to increase the difficulty of the implementation of endovascular therapy and can result in a patent FL (Qing et al., 2016). Future computational models should include multiple re-entry sites and dissection anatomies with rotated flaps to follow clinical observations. The present analysis serves as a foundation for future more realistic refinements of aortic dissection models.

## Author Contributions

AA is the first author of this research and contributed to this manuscript extensively. XG created dissections manually in the porcine samples, prepared them for bench testing, and assisted AA in conducting bench tests. JN prepared the tissue specimens and supervised the planar biaxial testing according to the protocol and assisted AA with the biomechanical characterization of tissues. JK provided expert insight into the material behavior of tissues and assisted with the calibration and functioning of bench testing machine. SH provided clinical inputs to the paper and contributed extensively to the section “Introduction” as well as in steering the objectives of this study. JN, BR, SC, and GK contributed to critical sections of the paper, the protocol, managing resources for bench tests and computational studies, and approving results for preparation of manuscripts.

## Conflict of Interest Statement

GK has received funding for this project from Cook Incorporated. The author list contains representatives from Cook Inc. that includes JN, JK, BR, and SC. The other authors declare that the research was conducted in the absence of any commercial or financial relationships that could be construed as a potential conflict of interest. The reviewer JB and handling Editor declared their shared affiliation.
