# Supplementary material for: Validated Computational Model to Compute Re-apposition Pressures for Treating Type-B Aortic Dissections
Source: Front Physiol. 2018 May 9;9:513. doi: 10.3389/fphys.2018.00513 (PMC5954206; doi:10.3389/fphys.2018.00513)
Supplement: Supplementary file 1 [file Data_Sheet_1.DOCX]

Supplementary Material

Appendix 1: Material characterization of tissue samples for computational modeling

**Aashish Ahuja^1^, Xiaomei Guo^1^, Jillian N. Noblet^2^, Joshua F. Krieger^2^, Blayne Roeder^2^, Stephan Haulon^3^, Sean Chambers^2^, Ghassan S. Kassab^1*^**

^1^California Medical Innovations Institute, San Diego, CA, USA

^2^Cook Medical, Bloomington, IN, USA

^3^Aortic Center, Hôpital Marie Lannelongue, Le pLessis Robinson, Université Paris Sud, France

****Correspondence*:** Ghassan S. Kassab; [gkassab@calmi2.org](mailto:gkassab@calmi2.org)

**§.1. Algorithm for non-linear regression modeling**

The non-linear regression techniques for determining the parameters of the HGO model were written in Python script. The data from multiple stretching protocols (1:1, 1:1.5, 1:2, 1.5:1, 2:1, with 1:1 being an equibiaxial loading condition) were used in the testing of tissues (Table 1). The following algorithm was proposed to select the best data to optimize the parameters for the HGO constitutive model:

1) Import the excel (or .csv) file that contained the load vs. displacement data recorded from the planar biaxial testing of the specimen.

2) Select the protocols (*i.e.,* 1:1, 1:1.5, 1:2, 1.5:1, 2:1 or all of them) which were utilized in curve fitting.

3) For each of the protocols, only select the region in the recorded data that correspond to tensile loading.

4) Since the planar biaxial data may contain noise while recording, we needed to filter the data. We used the Locally Weighted Scatterplot Smoothing (LOWESS) algorithm (Cleveland 1979) available in the statistical module of Python to remove noise from data.

5) After filtering the data for each protocol, we made several sets which covered data from each protocol as well as combinations of protocols. As an example, when we considered protocols, (1:1, 1:1.5, 1:2, 1.5:1, 2:1), we end up having 31 different data sets that contain 5 data sets considering each protocol, 10 data sets containing filtered data from with a combination of two protocols (*i.e.,* 1:1 & 1.5:1, 1:1 & 1:2, 2:1 & 1:1.5, *etc.*), 10 data sets containing filtered data from combination of three protocols (*i.e.* [1:1, 1:1.5, 1:2], [1:1.5, 1.5:1, 2:1], *etc.*), 5 data sets containing filtered data from combination of four protocols and 1 data set containing filtered data from all protocols.

6) Using Nelder-Mead minimization algorithm (Nelder and Mead 1965), we define the objective function considering an isochoric tissue (Equation 7). In this function, $\sigma_{\theta\theta}$ and $\sigma_{zz}$ are the Cauchy (true) stress data obtained from the experiments, $\sigma_{\theta\theta}^{\Psi}$ and $\sigma_{zz}^{\Psi}$ are the Cauchy stresses for the i^th^ point computed using Equation 7 and *n* is the number of data points. The minimization algorithm optimizes the five parameters for the HGO constitutive model, namely $C_{10}, k_{1}, k_{2}, \alpha\& \kappa$, using the objective function. The objective function is given as:

$\boldsymbol{\chi}^{\boldsymbol{2}}\boldsymbol{=}\sum_{\boldsymbol{i=1}}^{\boldsymbol{n}} \boldsymbol{[}\left( \boldsymbol{\sigma}_{\boldsymbol{\theta\theta}}\boldsymbol{-}\boldsymbol{\sigma}_{\boldsymbol{\theta\theta}}^{\boldsymbol{\Psi}} \right)_{\boldsymbol{i}}^{\boldsymbol{2}}\boldsymbol{+}\left( \boldsymbol{\sigma}_{\boldsymbol{zz}}\boldsymbol{-}\boldsymbol{\sigma}_{\boldsymbol{zz}}^{\boldsymbol{\Psi}} \right)_{\boldsymbol{i}}^{\boldsymbol{2}}\boldsymbol{]}$

The minimization problem is ill-conditioned and thus, has several solutions for given limits on parameters. To achieve a global minimum, the algorithm is repeated for 200 different initial values of the parameters. Only the parameter estimates corresponding to the lowest chi-square value is selected.

7) For every feasible solution we imposed conditions of $R^{2}\geq0.9$ and a mean square root error, $\epsilon\leq0.2$ that needs to be satisfied. The value of $\epsilon=\sqrt{\chi^{2}/(n-q)}/\varpi$ uses $\varpi=\sum_{i=1}^{n} \left( \sigma\right)_{i}/n$ as the mean value of the measured stresses, $q (=5)$ is the number of parameters optimized in the constitutive model so $(n-q)$ is the number of degrees of freedom.

8) Performing steps 1-7, we obtain parameter values for each considered combination of protocols. If the parameter values from each combination can fit the data given by the original protocols (*i.e.* 1:1, 1:1.5, 1:2, 1.5:1, 2:1) with a $R^{2}\geq0.8$ and a mean error value, $\epsilon\leq0.2$5, those parameter values were chosen.

9) Finally, the median of the parameter values from combinations of protocols satisfying Step 8 was computed. The median values for $C_{10}, k_{1}, k_{2}, \alpha\& \kappa$ were used to plot stress-strain curves for hyperelastic tissues.

10) In case the median of the parameter values obtained from Step 9 did not fulfil the criterion laid out in Step 8, we used the parameter values for the combination that considers data points from maximum number of protocols. This combination of protocols has already fulfilled the criterion in Step 8.

Table 1. Specifications of tissue sample and test protocols used for its material characterization

| Pig Number | Thickness of the tissue (mm) | Region of thoracic aorta and displacement-controlled protocols |
| --- | --- | --- |
| Pig 1 | 1. Mid TL wall: 1.76 2. Mid FL wall: 1.3 3. Distal FL wall: 1.24 4. Mid Flap: 0.58 5. Distal Flap: 0.4 | Mid TL wall (1:1), Mid Flap (1:1, 1:1.5, 1:2, 1.5:1, 2:1), Mid FL wall (1:1, 1:1.5, 1:2, 1.5:1, 2:1), Distal FL wall (1:1, 1.5:1, 2:1), Distal Flap (1:1, 1:1.5, 1:2, 1.5:1, 2:1) |
| Pig 2 | 1. Mid TL wall: 1.75 2. Mid FL wall: 1.06 3. Distal FL wall: 1.17 4. Mid Flap: 0.59 5. Distal Flap: 0.34 | Mid TL wall (1:1, 1:1.5, 1:2, 1.5:1, 2:1), Mid Flap (1:1, 1:1.5, 1:2, 1.5:1, 2:1), Mid FL wall (1:1, 1:1.5, 1:2, 1.5:1, 2:1), Distal FL wall (1:1, 1:2, 1.5:1, 2:1), Distal Flap (1:1, 1:1.5, 1:2, 1.5:1, 2:1) |
| Pig 3 | 1. Mid TL wall: 1.70 2. Mid FL wall: 1.04 3. Distal FL wall: 0.87 4. Mid Flap: 0.54 5. Distal Flap: 0.43 | Mid TL wall (1:1, 1:1.5, 1:2, 1.5:1, 2:1), Mid Flap (1:1, 1:1.5, 1:2), Mid FL wall (1:1, 1:1.5, 1:2, 1.5:1, 2:1), Distal FL wall (1:1), Distal Flap (1:1, 1:1.5, 1:2, 1.5:1, 2:1) |
| Pig 4 | 1. Mid TL wall: 1.75 2. Mid FL wall: 1.10 3. Distal FL wall: 0.85 4. Mid Flap: 0.70 5. Distal Flap: 0.29 | Mid TL wall (1:1, 1:1.5, 1:2, 1.5:1, 2:1), Mid Flap (1:1, 1:1.5, 1:2), Mid FL wall (1:1), Distal FL wall (1:1, 1:1.5, 1:2, 1.5:1, 2:1), Distal Flap (1:1, 1:1.5, 1:2, 1.5:1, 2:1) |
| Pig 5 | 1. Mid TL wall: 1.86 2. Mid FL wall: 1.33 3. Distal FL wall: 1.01 4. Mid Flap: 0.47 | Mid TL wall (1:1, 1:1.5, 1:2, 1.5:1), Mid Flap (1:1), Mid FL wall (1:1), Distal FL wall (1:1, 1.5:1, 2:1) |

Five samples from TL wall, mid and distal FL wall, mid flap regions and four samples from distal region were harvested. The planar biaxial testing of different tissue specimens yields the results summarized in Tables 2-6. The results in Fig. A1 show the stress-stretch curves along the circumferential and axial directions for tissue samples tested with an equibiaxial displacement-controlled protocol.

Table 2. Parameter Estimation for Mid True Lumen Wall

| Pig # | Thickness (mm) | C10 (Pa) | k_1_ (Pa) | k_2_ | α (deg) | κ |
| --- | --- | --- | --- | --- | --- | --- |
| 1 | 1.76 | 78,219 | 201,440 | 1.52 | 87.09 | 0.2 |
| 2 | 1.75 | 60,707 | 230,300 | 3.2 | 89.95 | 0.29 |
| 3 | 1.70 | 53,816 | 117,670 | 3.02 | 0 | 0.28 |
| 4 | 1.75 | 51,191 | 188,950 | 1.11 | 2.86 | 0.32 |
| 5 | 1.86 | 54,702 | 110,450 | 2.32 | 61.31 | 0.22 |

Table 3. Parameter Estimation for Mid False Lumen Wall

| Pig # | Thickness (mm) | C10 (Pa) | k_1_ (Pa) | k_2_ | α (deg) | κ |
| --- | --- | --- | --- | --- | --- | --- |
| 1 | 1.3 | 53,456 | 952,380 | 4.94 | 7.45 | 0.3 |
| 2 | 1.06 | 88,823 | 94,663 | 18.665 | 0.00 | 0.165 |
| 3 | 1.04 | 72,082 | 32,735 | 14.9 | 21.20 | 0.11 |
| 4 | 1.10 | 19,657 | 45,520 | 2.022 | 49.85 | 0 |
| 5 | 1.33 | 53,316 | 53,787 | 6.0417 | 0.80 | 0.22 |

Table 4. Parameter Estimation for Mid Flap

| Pig # | Thickness (mm) | C10 (Pa) | k_1_ (Pa) | k_2_ | α (degrees) | κ |
| --- | --- | --- | --- | --- | --- | --- |
| 1 | 0.58 | 92,963 | 230,290 | 13.90 | 87.1 | 0.33 |
| 2 | 0.59 | 73,144 | 235,075 | 7.86 | 68.7 | 0.3 |
| 3 | 0.54 | 64,042 | 212,120 | 4.99 | 23.5 | 0.32 |
| 4 | 0.70 | 52,072 | 125,430 | 5.87 | 53.9 | 0.26 |
| 5 | 0.47 | 45,588 | 149,880 | 1.42 | 55.6 | 0.21 |

Table 5. Parameter Estimation for Distal False Lumen Wall

| Pig # | Thickness (mm) | C10 (Pa) | k_1_ (Pa) | k_2_ | α (degrees) | κ |
| --- | --- | --- | --- | --- | --- | --- |
| 1 | 1.24 | 72,996 | 20,894 | 9.01 | 66.5 | 0 |
| 2 | 1.17 | 31,299 | 64,299 | 5.44 | 66.5 | 0.25 |
| 3 | 0.87 | 44,479 | 229,920 | 5.02 | 22.3 | 0.3 |
| 4 | 0.85 | 45,167 | 200,820 | 9.84 | 87.7 | 0.27 |
| 5 | 1.01 | 58,489 | 90,846 | 4.78 | 48.1 | 0.21 |

Table 6. Parameter Estimation for Distal Flap

| Pig # | Thickness (mm) | C10 (Pa) | k_1_ (Pa) | k_2_ | α (degrees) | κ |
| --- | --- | --- | --- | --- | --- | --- |
| 1 | 0.4 | 103,140 | 61,969 | 4.1 | 62.4 | 0.1 |
| 2 | 0.34 | 171,740 | 661,830 | 8.05 | 86.5 | 0.3 |
| 3 | 0.43 | 78,686 | 239,090 | 3.18 | 89.9 | 0.3 |
| 4 | 0.29 | 63,554 | 77,013 | 4.76 | 83.4 | 0.11 |


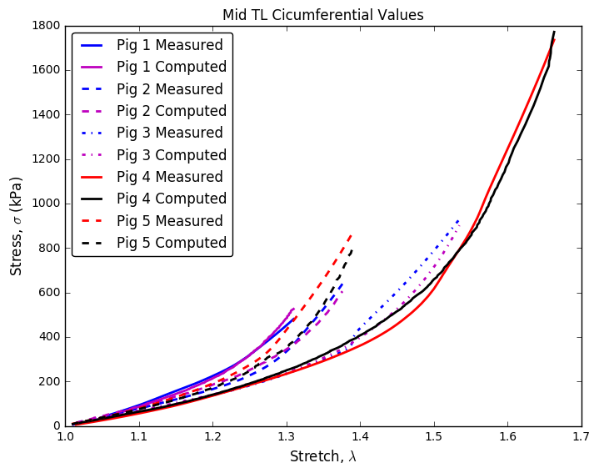

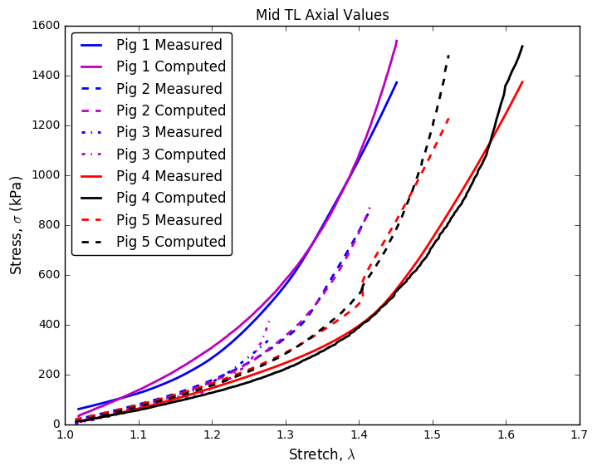


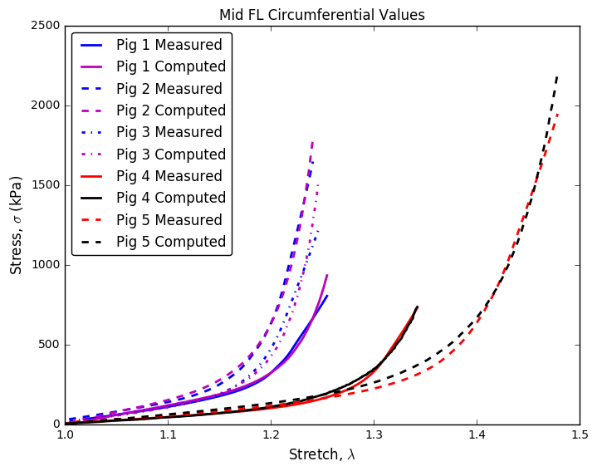

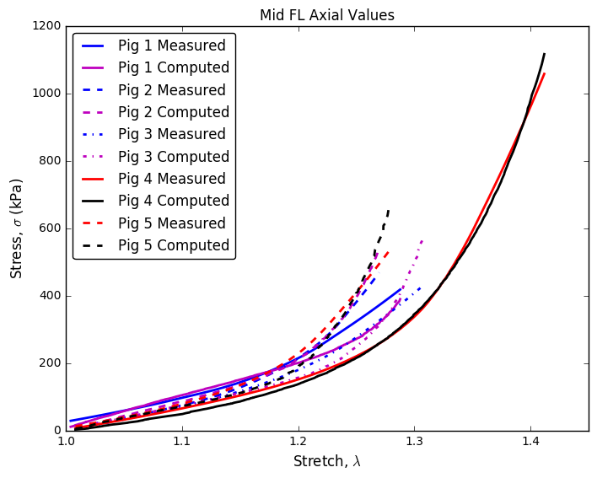


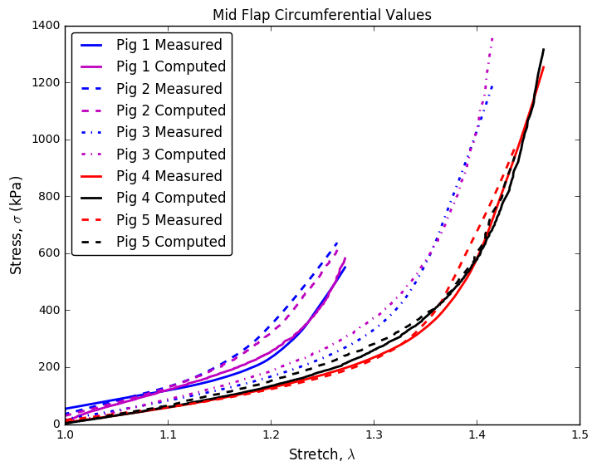

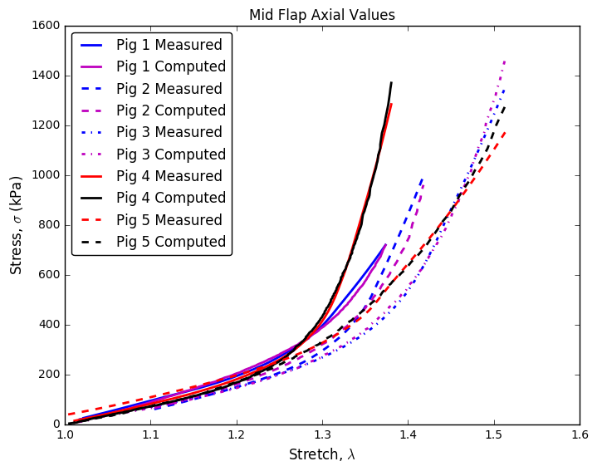


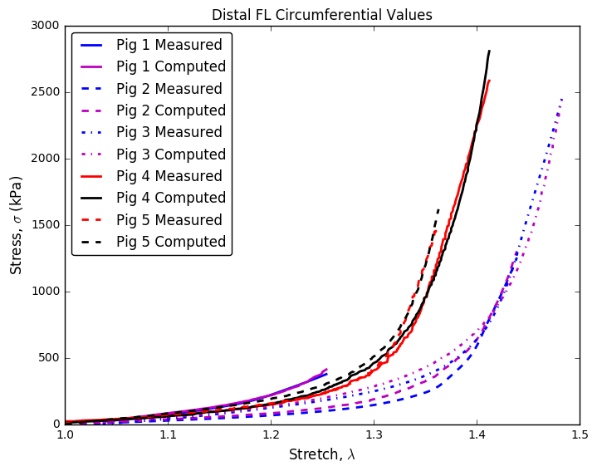

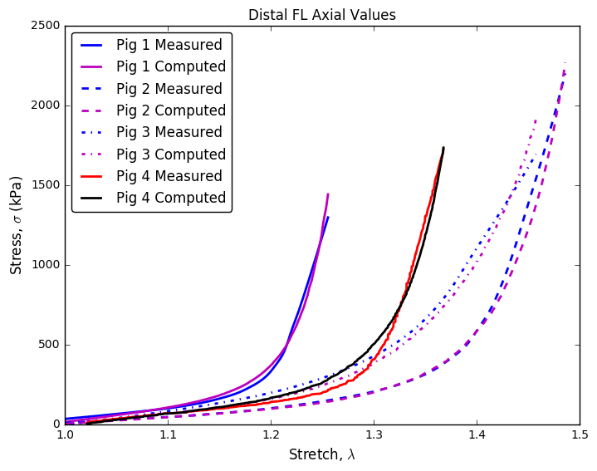


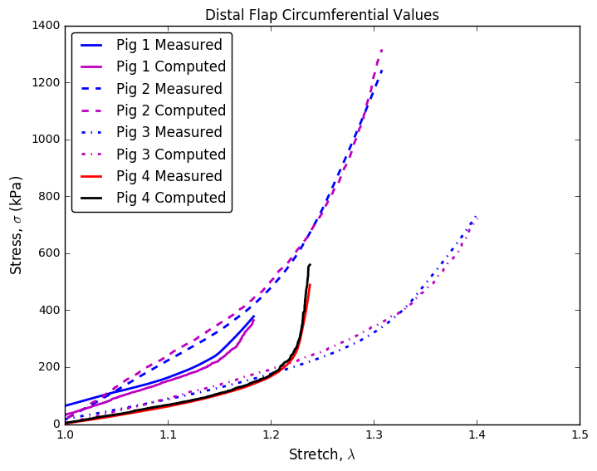

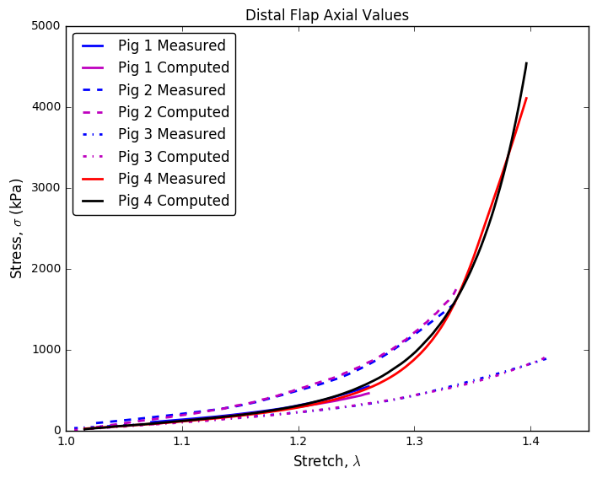


**Figure A1** Circumferential/Axial Stress vs Stretch relations for different regions of the dissected aorta.
